# Impact of frailty on periprocedural health care utilization in patients undergoing transcatheter edge-to-edge mitral valve repair

**DOI:** 10.1007/s00392-020-01789-5

**Published:** 2020-12-17

**Authors:** Christos Iliadis, Leandra Schwabe, Dirk Müller, Stephanie Stock, Stephan Baldus, Roman Pfister

**Affiliations:** 1 Department III of Internal Medicine, Faculty of Medicine and University Hospital Cologne, Heart Center, University of Cologne, Kerpener Strasse 62, 50937 Cologne, Germany; 2 Department of Diagnostic and Interventional Radiology, Faculty of Medicine and University Hospital Cologne, University of Cologne, Cologne, Germany; 3 Institute for Health Economics and Clinical Epidemiology, University of Cologne, Cologne, Germany

**Keywords:** MitraClip, Frailty, Hospital costs

## Abstract

**Background:**

Frailty is a common characteristic of patients undergoing transcatheter mitral valve repair (TMVR). It is unclear whether the physical vulnerability of frail patients translates into increased procedural health care utilization.

**Methods and results:**

Frailty was assessed using the Fried criteria in 229 patients undergoing TMVR using the MitraClip system at our institution and associations with total costs and costs by cost centers within the hospital incurred during periprocedural hospitalization were examined. Frail patients (*n* = 107, 47%) compared to non-frail patients showed significantly higher total costs [median/interquartile range, excluding implant costs: 7,337 € (5,911–9,814) vs 6,238 € (5,584–7,499), *p *= 0.001], with a difference in means of 2,317 €. Frailty was the only clinical baseline characteristic with significant association with total costs. Higher total costs in frail patients were attributable primarily to longer stay on intermediate/intensive care unit (3.8 ± 5.7 days in frail vs 2.1 ± 1.7 days in non-frail, *p *= 0.003), but also to costs of clinical chemistry and physiotherapy. The prolonged stay on intermediate/intensive care unit in frail patients was attributable to postprocedural complications such as bleeding, kidney injury, infections and cardiovascular instability.

**Conclusion:**

Frailty is associated with a mean 32% increase of hospital costs in patients undergoing TMVR, which is primarily the result of a prolonged recovery and increased vulnerability to complications. These findings are valuable for a hospital’s total cost calculation and resource allocation planning. Since frailty is regarded a potentially reversible health state, preventive interventions may help reduce costs in frail patients.

**Graphic abstract:**

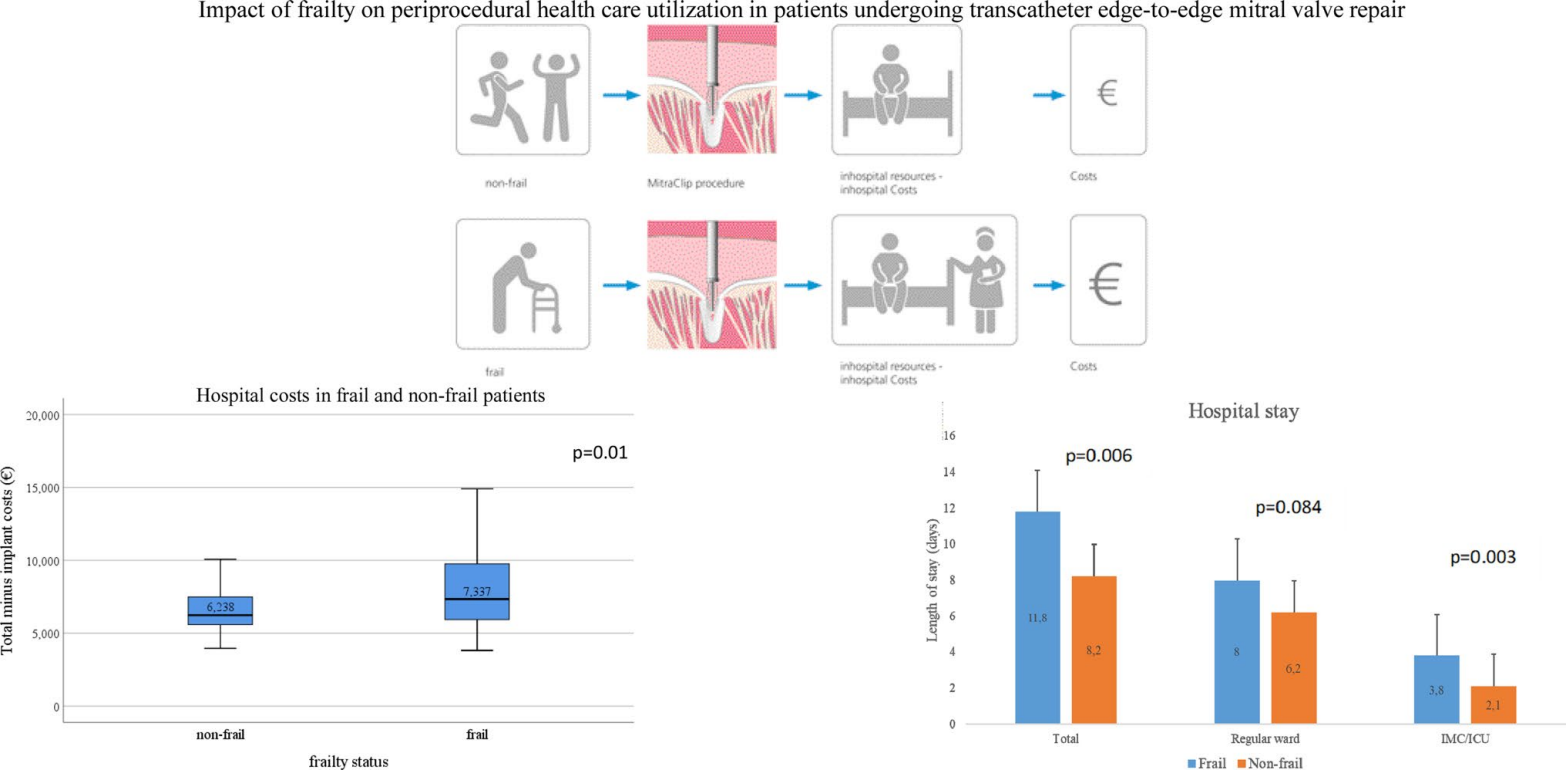

**Supplementary Information:**

The online version contains supplementary material available at 10.1007/s00392-020-01789-5.

## Introduction

The implications of frailty for the health care sector gain increasing attention in the context of ageing populations. Frailty is a complex clinical syndrome, which describes a decrease of physiological reserves that come along with an increased vulnerability to stressors [1]. Although frailty increases with aging, and is usually regarded as geriatric syndrome, it reflects a functional impairment beyond the chronological age [2]. For example, single severe organic disease or high cumulative comorbidity also contribute to the development of frailty [3].

Potential medical stressors where an increased vulnerability of frail people might become particularly relevant are surgery or interventional procedures. In patients undergoing percutaneous coronary intervention or cardiac surgery, frailty was associated with higher mortality, vascular and bleeding complications and renal failure [4–7]. These procedural complications translated into increased hospitalization costs in frail patients [8, 9]. Transcatheter valve interventions such as transcatheter aortic valve implantation [10] or transcatheter mitral valve repair (TMVR) were developed to decrease medical stress for patients [11, 12]. As consequence, this means that frailty is a frequent clinical reason to decide for a transcatheter approach and against surgical valve therapy and almost every second patient undergoing TAVI or TMVR is frail [13, 14].

So far it is unclear whether “low stress” procedures like TMVR have an impact on the complication rate, recovery from complications and resulting treatment costs in highly vulnerable patients. We have recently demonstrated that the risk of major cardiac or vascular complications was not increased in frail compared to non-frail patients undergoing TMVR [15]. However, major complications are overall very rare after TMVR and frailty might affect health care utilization through minor procedural or procedure independent complications following TMVR. The knowledge of determinants of treatment costs and underlying causes is of major relevance for all health care stakeholders. The aim of this study was to examine the association of frailty status with hospital related costs in consecutive patients undergoing TMVR with MitraClip at our institution.

## Methods

Consecutive adult patients undergoing TMVR using the MitraClip system (Abbott Vascular, Santa Clara, California, USA) at our institution between May 2014 and October 2016 were eligible for this retrospective analysis of prospectively assessed data on frailty in patients undergoing TMVR. Treatment decision for TMVR with MitraClip was taken by a multidisciplinary heart team after preoperative risk assessment based on objective risk scores, clinical parameters and morphological suitability for MitraClip according to current guidelines [16]. All patients underwent TMVR in general anesthesia. Extubation in the cath lab is pursued in every patient. By default, all patients are transferred to the cardiology intermediate care unit (IMC) for at least 24 h after the procedure. If medically indicated, monitoring on the IMC can be extended to more than 24 h, and patients could be transferred to the intensive care unit (ICU) post-procedurally if extubation was unsuccessful or in the case of severe haemodynamic compromise. Written informed consent for participation was obtained from all patients included in this study. The study was approved by the local ethics committee of the University of Cologne (reference 14-116, German Registry of Clinical Trials registration DRKS00006194).

Frailty status using the Fried criteria was prospectively assessed before the MitraClip procedure as reported earlier [15]. Briefly, the five components of the frailty syndrome (unintentional weight loss, weakness, exhaustion, slowness and low physical activity) were assessed, and 1 point was scored for each criterion met to specification. Patients meeting at least 3 of the 5 criteria were classified as frail. All treating physicians were blinded to the frailty status. For confirmatory analysis we used a second definition of frailty based on disability in Instrumental Activities of Daily Life (IADL) as recommended by the Mitral Valve Academic Research Consortium [17]. Disabilities in IADL have been shown to correlate with physical frailty in elderly people [18, 19]. Since a validated threshold of IADL for frailty definition is lacking, we pragmatically defined a frail status by the group median of the IADL score which corresponds to a disability in more than two IADL domains. Prespecified baseline clinical characteristics were extracted from the patient record. The reason for a prolonged IMC/ICU stay which we defined as more than two days was assessed retrospectively from records using predefined categories: (a) postprocedural minor or major bleeding according to MVARC (Mitral Valve Academic Research Consortium); (b) an acute kidney injury requiring haemodialysis; (c) an acute infection with antibiotic treatment; (d) haemodynamic instability which required inotropes or vasopressors, or arrhythmias leading to haemodynamic compromise; (e) respiratory failure with need of mechanical or non-invasive ventilation and (f) in absence of medical reasons logistic causes, meaning no vacancy to transfer the patient to the normal ward. All cost and reimbursement data were provided by the department of controlling of the University Hospital of Cologne. Total hospital costs were calculated as the sum of all incurred costs during hospital stay for elective TMVR from the day of hospital admission to discharge, including personnel expenses, material costs and costs for infrastructure. Personnel expenses for physician and nursing services, overhead costs for medication, other medical supply and medical and not-medical infrastructure were time based allocated to a patient. Revenues were calculated according to the German Diagnosis Related Groups (G-DRG) system on a case-based lump sum.

Mean and standard deviation or median and interquartile range (where data were not normally distributed) was used for continuous variables and frequency with percentage for categorical variables. *t* test (and Welch’s *t *test in case of unequal variances) or Mann–Whitney *U* test or Chi^2^-test was used for comparison between groups. All cost variables showed a highly skewed distribution and were described as median and interquartile range. However, we also present the difference in mean costs of the two groups which is interesting from the economic point of view. Since cost distribution was skewed, reciprocal transformation was used to achieve normal distribution. Univariable and multivariable linear regression analyses were applied to identify significant preoperative predictors of total costs minus implant costs. The statistical analyses were performed using IBM® SPSS® Statistics 25 (SPSS Inc., Chicago, Illinois, USA).

## Results

### Baseline characteristics

**Fig. 1:**
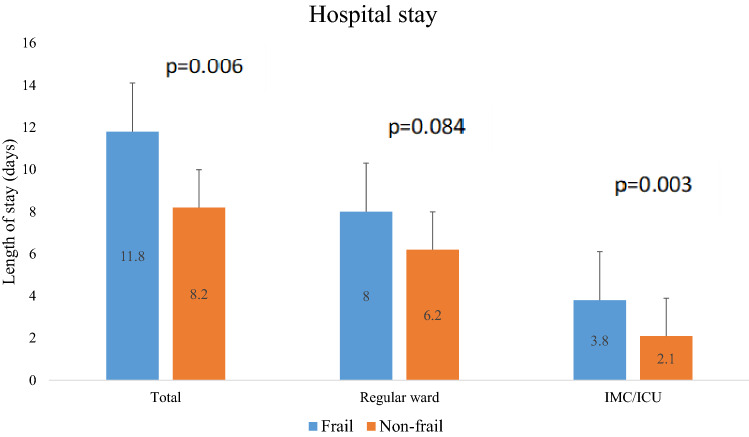
Days spent in the hospital: total, regular ward and intermediate/intensive care unit

229 patients (126 male and 103 female) with a mean age of 78 ± 8 years were included in the analysis and 46.7% were classified as frail according to Fried criteria. Table 1 shows the patients’ baseline characteristics. Frail patients were found to be of significantly higher age and had a worse functional status regarding NYHA functional class (NYHA class III or IV in 95% vs. 80% of patients) compared to non-frail patients. Frail patients had significantly higher EuroScore II and less previous cardiac surgery compared to non-frail patients (28% vs. 44%). Regarding sex and selected comorbidities—as reduced ejection fraction, diabetes mellitus or chronic obstructive pulmonary disease—there was no significant difference between the two groups. In contrary, frail patients had more severe renal impairment. Frailty according to Fried showed a significant association with frailty defined by disability in IADL. The difference in rate of urgent referrals was higher in frail patients which was of borderline significance. The total length of hospital stay and IMC/ICU stay were significantly increased in frail patients (Fig. 1).

**Table 1:** Comparison of clinical characteristics between frail and non-frail patients

	Frail (*n* = 107)	Non-frail (*n* = 122)	*p* value
Baseline			
Age, years	79 ± 7	77 ± 9	0.02
Male, *n* (%)	52 (49)	74 (61)	0.07
BMI (kg/m^2^)	25.6 ± 5.7	25.2 ± 5.6	0.45
Logistic Euroscore	22.1 ± 15.3	19.2 ± 15.2	0.09
Euroscore II	9.2 ± 7	7.4 ± 6.6	0.008
Secondary MR aetiology, *n* (%)	59 (55)	72 (59)	0.83
Hypertension, *n* (%)	80 (75)	89 (73)	0.76
Diabetes mellitus, *n* (%)	35 (33)	27 (22)	0.07
Previous stroke/TIA, *n* (%)	13 (12)	17 (14)	0.69
Previous myocardial infarction, *n* (%)	33 (31)	35 (29)	0.72
Coronary artery disease, *n* (%)	65 (61)	73 (60)	0.89
Previous cardiac surgery, *n* (%)	30 (28)	54 (44)	0.01
Previous ICD, *n* (%)	16 (15)	27 (22)	0.17
Previous CRT, *n* (%)	24 (22)	21 (17)	0.32
PAOD, *n* (%)	13 (12)	13 (11)	0.72
COPD, *n* (%)	19 (18)	18 (15)	0.52
Atrial fibrillation, *n* (%)	70 (65)	68 (56)	0.14
Malignancy, *n* (%)	16 (15)	21 (17)	0.64
GFR (ml/min/1.73m^2^)	41.6 ± 20	51.2 ± 20	0.001
EF > 50%, *n* (%)	56 (52)	54 (44)	0.4
NYHA, *n* (%)			< 0.001
I/II	5 (5)	24 (20)	
III/IV	102 (95)	98 (80)	
Frailty by IADL disability	71 (66.4%)	40 (32.8%)	< 0.001
Urgent referral status, *n* (%)	18 (17)	10 (8)	0.047
Total length of stay (days)	11.8 ± 9.7	8.2 ± 9.9	< 0.001
Stay on regular ward	8.0 ± 7.3	6.2 ± 8.7	0.03
Stay on intensive/intermediate care unit	3.8 ± 5.7	2.1 ± 1.7	< 0.001

Data are presented as arithmetic mean and standard deviation or frequency and percentage. Comparison between groups by *t *test or Chi^2^-test

*BMI* body-mass index, *COPD* chronic obstructive pulmonary disease, *EF* ejection fraction, *GFR* glomerular filtration rate, *IADL* instrumental activities of daily life, *MR* mitral regurgitation, *MVARC* mitral valve academic research consortium, *NYHA* New York heart association, *PAOD* peripheral artery occlusive disease, *TIA* transient ischemic attack

### Costs and revenues

**Fig. 2:**
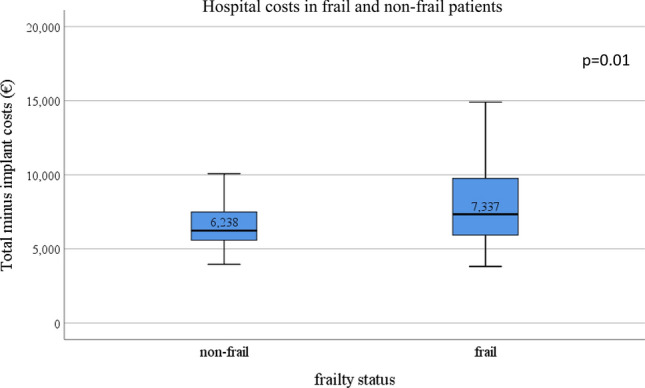
Hospital costs in frail and non-frail patients

Frail patients showed significantly higher total costs [28,225 € (26,832–31,756)] compared to non-frail patients [27,459 € (26,457–28,377), *p *= 0.002, Table 2]. Since the implant (MitraClip device) was by far the largest cost item (21,000 €, 72% of all costs) an additional analysis was performed, focusing on total costs excluding implant costs. Still, costs were significantly increased in frail patients [7,337 € (5,911–9,814)] compared to non-frail patients [6,238 € (5,584–7,499)] (*p *= 0.001), with a difference of means of 2,317 € (Fig. 2). The revenues were significantly different between frail and non-frail patients [31,959 € (31,825–32,954)] vs. [31,825 € (31,638–32,834), *p *= 0.034], with a difference of means of 99 €. In 22.4% of cases, the revenue was not cost-covering in frail patients compared to 6.6% in non-frail patients (*p *= 0.001).

**Table 2:** Comparison of hospital costs by operated units and revenue between frail and non-frail patients

Hospital costs (€)	Frail (*n* = 107)	Non-frail (*n* = 122)	*p* value
Total costs	28,225 (26,832–31,756) [30,712 ± 7,754]	27,459 (26,457–28,377) [28,240 ± 4,467]	0.002
Total costs minus implant costs	7,337 (5,911–9,814) [9,449 ± 7,188]	6,238 (5,584–7,499) [7,132 ± 4,344]	0.001
Regular ward	1,670 (1,135–2,543) [2,258 ± 1,902]	1,404 (980–1,843) [1,746 ± 2,274]	0.011
Intermediate/intensive care unit	1,401 (863–2,468) [2,973 ± 5,057]	1,294 (746–1,459) [1,438 ± 1,269]	0.001
Anaesthesia	570 (457–706) [605 ± 368]	568 (463–727) [600 ± 192]	0.671
Cardiac catheter laboratory^a^	23,931 (22,935–24,403) [23,867 ± 1,700]	23,821 (23,185–24,450) [23,867 ± 1,222]	0.931
Radiology	28 (13–75) [90 ± 154]	26 (0–50) [97 ± 230]	0.049
Clinical chemistry	223 (161–422) [385 ± 451]	173 (140–239) [263 ± 302]	0.002
Other diagnostics	141 (93–288) [188 ± 134]	108 (93–242) [160 ± 125]	0.092
Physiotherapy	31 (6–77) [101 ± 229]	9 (0–35) [31 ± 58]	0.034
Revenues	31,959 (31,825–32,954) [32,964 ± 8,208]	31,825 (31,638–32,834) [32,865 ± 10,185]	0.034

Data are presented as median and interquartile range [mean ± standard deviation] Comparison between groups by Mann–Whitney-*U* test

*BMI* body-mass index, *COPD* chronic obstructive pulmonary disease, *EF* ejection fraction, *GFR* glomerular filtration rate, *IADL* instrumental activities of daily life, *MR* mitral regurgitation, *MVARC* mitral valve academic research consortium, *NYHA* New York heart association, *PAOD* peripheral artery occlusive disease, *TIA* transient ischemic attack

^a^Includes the implant cost

All baseline characteristics of Table 1 were examined in univariate linear regression with total minus implant costs as dependent variable. All significant variables of univariate analysis (Euroscore II, frailty, glomerular filtration rate and urgent referral status) were examined in a multivariate model. Frailty remained a significant factor in predicting total costs minus implant costs (*p *= 0.04 for frailty in linear regression analysis).

When defining frailty by disability in IADL, results were virtually the same. Patients with disability had significantly higher total costs [27,937 € (26,832–30,505) vs. 27,401 € (26,428–29,097), *p *= 0.03] and significantly higher total costs excluding implant costs [7,080 € (5,879–9,105) vs. 6,351 € (5,611–7,812), *p *= 0.02].

To evaluate the impact of cost outliers, the total cost analysis was repeated after excluding high cost cases (total costs > 40,000 €). Cost outliers accounted for 4% of the total study population. Considerably high costs occurred in patients with urgent MitraClip with cardiac decompensation and prolonged hospital stay due to the need for recompensation; prolonged respiratory failure with need for respiratory support; infection leading to severe sepsis with need for intensive medical care; and severe kidney injury, often as the manifestation of a cardio-renal syndrome, with need for repeated hemodialysis. Clinical details of the nine cases which were cost outliers are described in Supplementary Table 1. These cases were associated with an elaborate intensive medical therapy which accounted for considerably higher costs.

The higher total costs in frail patients were attributable to significant differences in costs incurred on IMC/ICU, on regular ward, for physiotherapy and laboratory services. The higher costs incurred on IMC/ICU and regular ward (personnel expenses for physician and nursing services, overhead costs for medication, other medical supply and medical and not-medical infrastructure) are time based allocated to a patient and therefore expected to increase with a prolonged stay.

Since costs and revenues might change over time, stratified analysis was performed by year of procedure performed. There was a trend for a decrease of total costs and total costs minus implant costs from the year 2014 to the year 2016 and no obvious temporal trend of revenues (Supplementary Table 2). The results of the total population with higher costs in frail patients and virtually no relevant difference of revenues between frail and non-frail patients was found in all 3 years of the study observation.

### Prolonged IMC/ICU stay

The rate of patients with a prolonged IMC/ICU stay was significantly increased in frail (*n* = 38, 36%) compared to non-frail patients (*n* = 23, 19%, *p *= 0.04). The causes of a prolonged IMC/ICU stay are listed in Table 3. All post-procedural complications associated with a prolonged stay were more frequent in frail patients.

**Table 3:** Comparison of causes for prolonged stay. Data are presented as frequency and percentage

	*n* = 38/107 frail patients	*n* = 23/122 non-frail patients
MVARC major or minor bleeding	14 (13.1%)	7 (5.7%)
Acute kidney injury requiring haemodialysis	3 (2.8%)	0 (0%)
Infection	4 (3.7%)	2 (1.6%)
Haemodynamic instability/arrhythmias	6 (5.6%)	5 (4.1%)
Respiratory failure	4 (3.7%)	4 (3.3%)
Logistics/other	7 (6.5%)	5 (4.1%)

*BMI* body-mass index, *COPD* chronic obstructive pulmonary disease, *EF* ejection fraction, *GFR* glomerular filtration rate, *IADL* instrumental activities of daily life, *MR* mitral regurgitation, *MVARC* mitral valve academic research consortium, *NYHA* New York heart association, *PAOD* peripheral artery occlusive disease, *TIA* transient ischemic attack

## Discussion

Frailty is a leading cause for high surgical risk and the decision for a TMVR approach in patients with mitral regurgitation. Although about half of patients undergoing TMVR are frail [14, 15, 20], data on the impact of frailty on procedural health care utilization is lacking. Mean total costs without implant expenses were 32% higher in frail patients, and the association between frailty and costs persisted after excluding high cost outliers and was consistent across different definitions of frailty. Importantly, frailty was the only significant predictor of total costs when considering baseline patient characteristics including sex, age, comorbidities, NYHA functional class and history of previous cardiac surgery. The increase in hospital costs in frail patients was primarily the result of a prolonged stay on the IMC/ICU caused by minor medical complications.

Our data extend existing evidence on the role of frailty for adverse outcomes in various health care settings. A body of evidence exists on the effect of frailty on prolonged recovery time after surgical procedures; in particular, a higher rate of institutionalization, increased mortality [21, 22] and the disproportionally high health care utilization associated with frailty in industrialized countries overall [23, 24]. Here, we show the necessity of prolonged postprocedural IMC/ICU and total hospital stay and associated increase in costs in the TMVR population. TMVR is a low stress intervention particularly developed for frail patients. In the setting of transcatheter aortic valve implantation [10], which is another minimal invasive procedure, frailty was a cost driver due to a prolonged length of stay, a high rate of complications and institutional discharge [25]. However, these results cannot be directly translated to the setting of TMVR. First, the burden of heart failure is higher in the TMVR population. Patients with severe aortic stenosis undergo a TAVI soon after symptoms arise, whereas mitral regurgitation (in particular secondary MR) is commonly associated with left ventricular failure and consequently these patients receive appropriate treatment (TMVR) after having already suffered from long-term symptomatic heart failure. Second, procedural complications are rare in the setting of TMVR and most often are not critical, whereas TAVI has the potential of early life-limiting complications, which occur more frequently in frail patients [26].

We have shown recently that MitraClip procedure shows similar technical and procedural efficacy in frail and non-frail patients [15]. Major cardiac structural and vascular access site complications were also not different. So, what might be the reason for the increased length of stay on IMC/ICU and regular wards? Frail patients were older, had a worse NYHA class and were generally sicker as reflected by the higher EuroScore II. However, frailty was the only predictor of hospital costs and comorbidities were not. A detailed analysis of underlying reasons for the prolonged hospital stay is beyond the scope of this study and partly impossible in a retrospective design since total hospital stay is the result of a complex interplay between medial, social, functional and logistic determinants. However, frail patients caused higher costs for laboratory services and physiotherapy, which are based on direct patient level data. Although these cost centre groups account for only a small absolute cost difference, these findings suggest that frail patients for example need more support to regain mobility/physical capabilities than non-frail patients. In support of the latter, frail patients have substantially worse disease independent measures of physical functioning both before and after TMVR than non-frail patients [15]. Furthermore, our analysis of medical causes of prolonged IMC/ICU stay demonstrated more minor complications or complications not directly associated with the TMVR procedure. Clinically severe complications such as haemodynamic compromise and dialysis might be generally more frequent in frail patients due to the higher baseline cardiac and extracardiac morbidity. For instance, baseline glomerular filtration rate was lower in frail patients which increases the risk of postprocedural renal failure. Prolonged respiratory failure and mechanical ventilation can be also explained by reduced cardiopulmonary reserve in frail patients but might also be attributable to muscle weakness and sarcopenia which are typical characteristics in frail patients [21]. We do not know whether the incidence of complications such as minor bleeding or infections overall are higher, or whether these complications are only more relevant for the clinical course of frail compared to non-frail patients. Such complications are usually only documented if clinically relevant or manifest and as such could not be systematically assessed in our retrospective analysis. However, considering the pathophysiological concept of frailty as increased vulnerability to stressors it seems plausible that mild bleeding anemia or inflammatory response might contribute to delayed recovery of frail patients and in consequence to longer hospital stay and higher costs [27].

Our findings have several important implications. First, from a methodological point of view cost analysis provides complementary data of patient outcomes to primary medical data. This is of major interest since frequency of complications might not differ in frail and non-frail patients but might have different impact on outcome and health care utilization. For instance, in patients undergoing TAVI there was a significant interaction between frailty status and access site on clinical outcome demonstrating that increased stress of transapical access was only of prognostic relevance in vulnerable, frail patients [28]. Second, frailty has a substantial impact on reimbursement of TMVR patients. Although on average the revenues exceeded the costs in frail and non-frail patients, the reimbursement was not covering costs in only 7% of non-frail patients and in 22% of frail patients. This knowledge is valuable for a hospital’s total cost calculation and resource allocation planning. Given the expected increase of frailty in the aging population, it should be considered to integrate frailty as a diagnosis determining case complexity and hence magnitude of revenue within the DRG based health care system. Our findings must not be interpreted as to deny TMVR in frail patients because of increased costs. In contrast, we observed a more pronounced symptomatic benefit of frail compared to non-frail patients after TMVR indicating a similar net cost-effectiveness. Third, physical frailty is regarded a potentially reversible health state. As such, the implementation of a frailty screening prior intervention and a tailored approach to disabilities of frail patients with early interventions during or even prior to the hospital stay may reduce length of stay and total costs in frail patients [29]. A positive impact of physical exercise, nutritional supplement and cognitive training on functional outcome has been reported, and geriatric-specific care protocols are discussed to shorten hospital length of stay in frail patients [30]. As TMVR compared to medical therapy alone has shown to result in a lower rate of hospitalization for heart failure and lower all-cause mortality within 24 months of follow-up in highly selected patients [31], further research should evaluate the cost-effectiveness or cost-utility of TMVR with regard to frailty.

### Study limitations

When interpreting the data, it must be considered that prices, cost calculation and reimbursement systems differ worldwide, and thus absolute estimates may not be generalizable from the German DRG-based system to other health care systems. For example, the length of hospital stay after TMVR is longer in Germany [median 9 (6–15) days] than in the US [median 2 (1–5) days] [6, 7]. However, the underlying medical context of frailty with increased resource utilization after TMVR and associated costs as well as our findings on medical reasons for prolonged ICU/IMC stay will be generally applicable. Furthermore, due to the retrospective study design it was not possible to determine individual reasons for prolonged hospital stay and increased costs. With respect to the latter, is must be emphasized that costs associated with the centre groups of IMC/ICU and regular ward are not derived from patient level data but are estimations based on time spent.

In order to estimate the resource use, data on costs was based on a case-based lump sum (i.e., G-DRG) which did not accurately reflect the real net effect of frailty on incurred costs. However, calculations based on individual patient data would have required a different study design applying a detailed process of cost calculation which was beyond the scope of this analysis.

## Conclusion

Frailty is associated with a mean 32% increase of hospital costs in patients undergoing TMVR, which is primarily the result of a prolonged recovery and increased vulnerability to complications. These findings are valuable for a hospital’s total cost calculation and resource allocation planning. Since frailty is regarded a potentially reversible health state, preventive interventions may help reduce costs in frail patients.

## Supplementary Information

Below is the link to the electronic supplementary material.

Supplementary file1 (DOCX 19 KB)
