# Supplementary material for: Impact of frailty on periprocedural health care utilization in patients undergoing transcatheter edge-to-edge mitral valve repair
Source: Clin Res Cardiol. 2020 Dec 17;110(5):658–66. doi: 10.1007/s00392-020-01789-5 (PMC8099800; doi:10.1007/s00392-020-01789-5)
Supplement: Supplementary file 1 — Supplementary file1 (DOCX 19 KB) [file 392_2020_1789_MOESM1_ESM.docx]

Supplementary Table 1. Cost outliers (N=9) in the study sample and the reasons for prolonged stay.

| Cases | Reasons for prolonged stay | Costs (€) |
| --- | --- | --- |
| 1 | Urgent referral from nephrology department due to decompensation, acute renal failure with need for new haemodialysis, urgent intervention due to refractory decompensation. After intervention stroke and complex rehabilitation including logotherapy | 40,737 |
| 2 | Urgent referral from emergency department due to decompensation and urgent intervention due to recurrent heart failure. After intervention bleeding with need for transfusion, long stay on ICU due to multiple organ failure | 77,352 |
| 3 | After intervention acute renal failure with need of new haemodialysis and septic shock | 45,881 |
| 4 | Urgent referral from emergency department due to decompensation and urgent intervention due to refractory heart failure. After intervention delirium and septic shock | 43,858 |
| 5 | Urgent referral from other hospital due to decompensation, delirium and pneumonia, need for mechanical ventilation, urgent intervention due to pulmonary oedema. After intervention ventricular tachycardia with haemodynamic compromise and need for intubation. Logotherapy due to recurrent aspiration risk | 59,314 |
| 6 | Urgent referral from other hospital due to decompensation and acute renal failure. Urgent intervention due to refractory heart and renal failure. After intervention, prolonged awakening phase due to uraemia or hepatic encephalopathy with need for monitoring | 64,851 |
| 7 | Chronic cardiac low output due to severe heart failure, long stay on intermediate care station for high-urgency heart transplantation listing. The transplantation listing was discontinued due to failure of listing requirements. Urgent intervention due to refractory heart failure with pulmonary oedema | 69,505 |
| 8 | After intervention sepsis due to leg ulcera and gangrene, urgent femoral-popliteal bypass grafting, septic shock | 46,109 |
| 9 | Urgent referral from emergency department due to decompensation and urgent intervention due to refractory heart failure with mechanical ventilation due to pulmonary oedema. After intervention severe skin infection with antibiotic treatment and neurological disorders | 40,016 |

Supplementary Table 2. Comparison of costs and revenues by year of treatment between frail and non-frail patients. Data are presented as median and interquartile range [mean ± standard deviation] Comparison between groups by Mann-Whitney-U test.

| Hospital costs (€) | Frail (n=107) | Non-Frail (n=122) | p-value |
| --- | --- | --- | --- |
| Total costs |  |  |  |
| Year 2014 (N=46) | 29,188 (27,697-30,818)  [31,790 ± 10,614] | 27,733 (27,240-28,996)  [28,275 ± 1,729] | 0.03 |
| Year 2015 (N=94) | 28,860 (27,480-29,166)  [31,204-6,617] | 27,711 (26,924-29,166)  [28,318-2,322] | 0.02 |
| Year 2016 (N=89) | 27,241 (25,759-30,925)  [29,747 ± 7,108] | 26,491 (25,750-27,654)  [28,124 ± 6,920] | 0.16 |
| Total costs minus implant costs |  |  |  |
| Year 2014 (N=46) | 7,788 (6,297-9,418)  [10,035 ± 9,029] | 6,333 (5,840-7,596)  [6,875 ± 1,729] | 0.03 |
| Year 2015 (N=94) | 7,460 (6,080-11,357)  [9,730 ± 6,544] | 6,311 (5,524-7,766)  [6,928 ± 2,322] | 0.02 |
| Year 2016 (N=89) | 6,675 (5,385-9,353)  [8,913 ± 6,860] | 6,105 (5,355-7,229)  [7,534 ± 6,677] | 0.16 |
| Revenues |  |  |  |
| Year 2014 (N=46) | 33,196 (31,638-33,600)  [34,688 ± 9,347] | 31,638 (31,638-31,638)  [31,973 ± 751] | 0.02 |
| Year 2015 (N=94) | 31,825 (31,825-31,825)  [32,681 ± 4,784] | 31,825 (31,825-31,825)  [31,701 ± 478] | 0.35 |
| Year 2016 (N=89) | 32,834 (31,959-32,894)  [32,372 ± 9,920] | 32,834 (32,834-32,834)  [34,780 ± 16,894] | 0.83 |
